# Cellulose Nanocrystals for Advanced Optics and Electronics: Current Status and Future Directions

**DOI:** 10.3390/mi16080860

**Published:** 2025-07-26

**Authors:** Hyeongbae Jeon, Kyeong Keun Oh, Minkyu Kim

**Affiliations:** Department of Chemical Engineering, Dankook University, Yongin 16890, Republic of Korea; hbj72240186@dankook.ac.kr

**Keywords:** cellulose nanocrystals, chirality, optics, electronics, good health and well-being

## Abstract

Cellulose nanocrystals (CNCs) have attracted growing interest in optics and electronics, extending beyond their traditional applications. They are considered key materials due to their fast computing, sensing adhesion, and emission of circularly polarized luminescence with high dissymmetry factors. This interest arises from their unique chemical structure, which gives rise to structural color, a chiral nematic phase, and high mechanical strength. In this perspective, we first introduce the definition, sources, and fundamental properties of CNCs to explain the basis for their unique and effective use in optics and electronics. Next, we review recent research on the application of CNCs in these fields. We then analyze the current limitations that hinder further advancement. Finally, we offer our own perspective on future directions for the CNC-enabled advanced optics and electronics.

## 1. Introduction

Numerous biomaterials possess fascinating functionalities. For instance, keratin-based hierarchical structures exhibit multifunctionality and mechanical resilience, playing essential roles such as defense, stress signaling, courtship display through structural coloration, and thermal insulation [1]. Additionally, 3,4-dihydroxyphenylalanine (DOPA), a well-studied key component responsible for mussel adhesion, is widely recognized for its remarkable ability to enhance polymer adhesion, even in wet environments [2]. As the most abundant organic polymer on Earth, cellulose nanocrystals (CNCs) also exhibit a range of appealing multifunctional properties, including biodegradability, sustainability, high specific surface area, high mechanical strength, potential for diverse surface modifications, structural color, and chirality [3]. These characteristics have enabled the development of novel materials for various advanced applications in optics, electronics, energy systems, and catalysis [3,4,5,6]. From this perspective, we first provide a comprehensive overview of the fundamental properties of CNCs and then focus on their contributions to advancements in optics and electronics. Finally, based on our analysis, we propose future directions for CNCs to further advance these fields.

## 2. Features of CNCs

### 2.1. Source of CNCs

**Figure 1:**
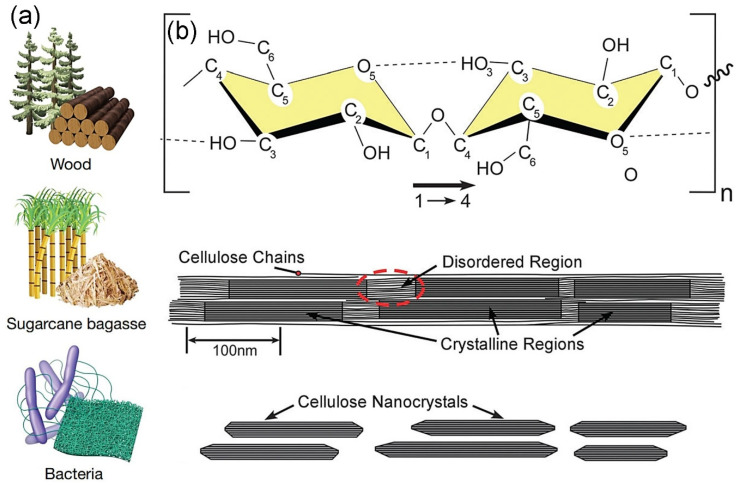
(**a**) Diverse precursors to produce cellulose (reprint from Ref. [7]). (**b**) Illustrations of a repeating unit of a cellulose chain (top panel), a model representation of a cellulose microfibril depicting a proposed arrangement of crystalline and amorphous domains (middle panel), and cellulose nanocrystals formed through acid hydrolysis, selectively removing the disordered regions (bottom panel) (reprint from Ref. [10]).

As a natural polysaccharide, cellulose acts as a key structural component in the primary cell walls of plants, various types of algae, oomycetes, and tunicate animals (Figure 1a). Additionally, certain bacteria such as *Medusomyces gisevii* and *Komagataeibacter xylinus* produce cellulose to create biofilms [7,8]. In other words, we can extract cellulose from these plants, algae, oomycetes, and tunicate animals. Currently, wood pulp is a mainstream source in cellulose synthesis [9]. This is because, in addition to the abundance of wood, the infrastructure for producing cellulose is most well-established for wood pulp compared to other sources, which is due to long-lasting and diverse applications of cellulose prepared from wood pulp, such as paper production, packaging materials, and capsules containing medicines [10]. Chemically, cellulose is composed of a long and unbranched chain of D-glucose molecules connected by β (1→4) glycosidic bonds as shown in Figure 1b [3,10,11]. In terms of its crystalline structure, cellulose is composed of crystalline and amorphous regions [3]. When the amorphous regions are hydrolyzed using strong acids such as sulfuric acid, the remaining crystalline regions yield CNCs [12].

### 2.2. Properties of CNCs Utilized for Optics and Electronics

**Figure 2:**
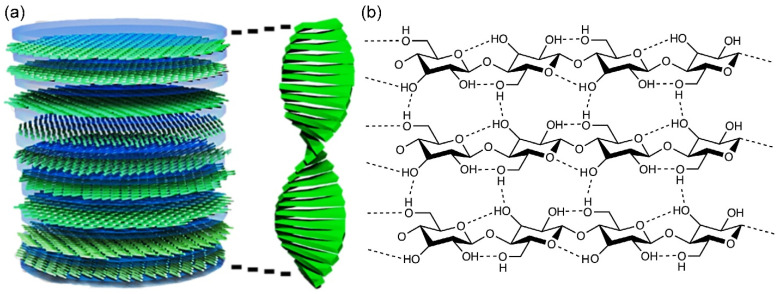
(**a**) Schematic for self-assembled twisted needle-like CNCs with left-handed chiral nematic structure (reprint from Ref. [13]). (**b**) Cellulose fibers exhibit aligned polymer chains, with intra- and intermolecular hydrogen bonding contributing to their cohesive structural organization (reprint from Ref. [11]).

As described above, the multifunctional properties of CNCs enable their use in advanced optical and electronic applications. Then, what are the underlying principles that render CNCs to exhibit such multifunctional properties? The answer lies in their unique chemical structure. As illustrated in Figure 2, the basic unit for CNC is D-glucose that has chirality at the atomic scale. When this D-glucose unit is covalently linked by β (1→4) glycosidic bonds, a single polymer chain is formed. If this single polymer chain is interconnected in ordered manner, crystalline CNC with twisted needle-like chiral structure is generated at nanoscale. Finally, self-assembled CNCs present left-handed chiral nematic structure at micro-scale [13,14]. Based on this principle, CNCs can show three-level hierarchical chirality: atom-, nano-, and micro-scale. What is more, thanks to the repeating distance between each nematic layer in chiral nematic structure, the Bragg reflection occurs, leading to generation of structural color. Furthermore, left-handed circular polarized light is reflected by left-handed chiral nematic structure of self-assembled CNC film. In addition, due to the abundant hydrogen bonds between the D-glucose unit within individual CNC needle, it can show extremely high mechanical properties, e.g., 105 GPa of elastic modulus in axial direction [11,15]. Ultimately, the rich-hydrogen bond between CNC needles leads to high tensile strength of 46.2 ± 4.8 MPa of CNC film, which is as high as that of metals [11,16].

## 3. Optical and Electronic Applications of CNCs

As described above, self-assembled CNCs are capable of not only producing structural colors but also selectively reflecting left-handed circularly polarized light. This fundamental property of self-assembled CNCs has led to its advanced optical and electronic applications including light controllers and optical sensors, as presented below.

### 3.1. Light Controllers

Light control is of great importance in the myriad fields. For instance, the emission of amplified circularly polarized light is of significance in quantum optical information and science [17]. The control of the lights by CNCs can be classified into the following three big categories: controlling the direction of the (1) transmitted lights, (2) reflected lights, and (3) emitted lights.

#### 3.1.1. Control of Transmitted Lights

**Figure 3:**
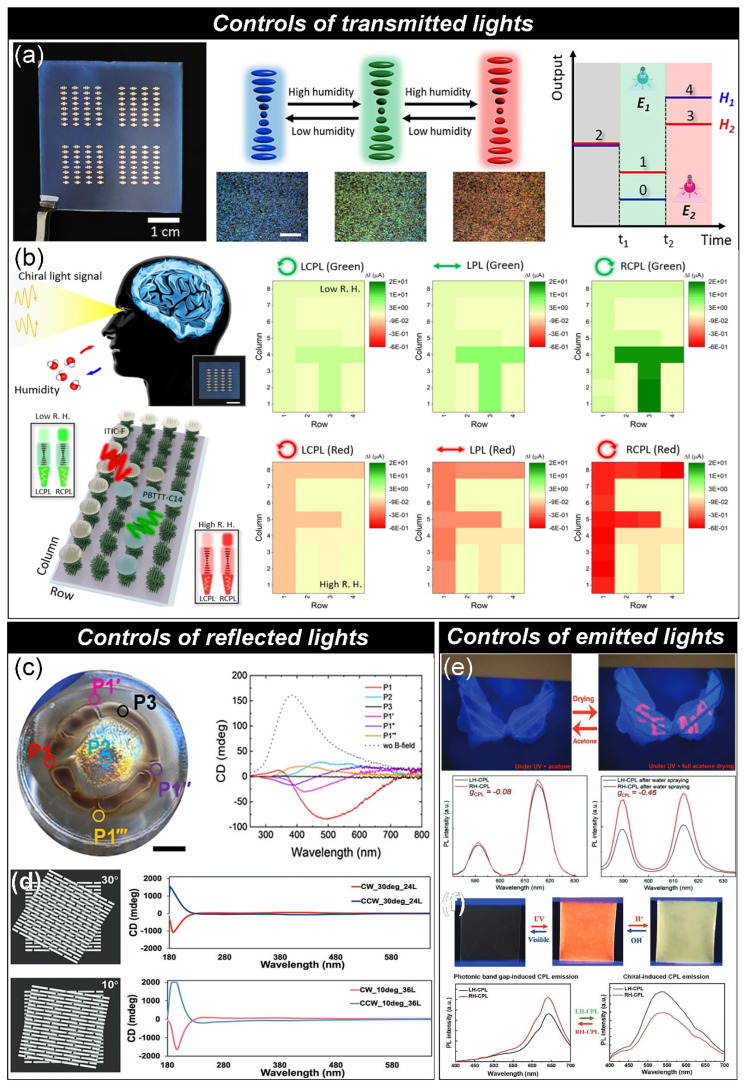
(**a**) A multivalued logic element for organic thin-film transistor. Photo of the CNC−based organic thin−film transistor arrays on 5 × 5 cm^2^ sized indium tin oxide glass used as substrate (left). The CNC/polyethylene glycol/NaCl composite film which changes its structural color in response to the humidity as presented in optical microscope images (middle) The scalebar in the optical microscope images presents 200 μm. Illustration showing principle of the reconfigurable ternary system based on the CNC composite film: output voltage changes in respond to photonic bandgap (H) and photon energy (E) (right). (reprint from Ref. [18]). (**b**) Left: schematic for recognition of the inkjet−printed signs by modulating wavelength and polarized state of the applied lights with changing relative humidity by CNC/polyethylene glycol/NaCl composite. The scale bar of inset photograph presents 1 cm. T (right top) and F (right bottom) are differently recognized by changing the wavelength and polarized state of the applied lights under application of electricity. (reprint from Ref. [19]). (**c**) Photo of the CNC/Fe_3_O_4_ composite film dried on a magnetic pattern (left). Various positions, labeled with different letters, were selected for measuring chiroptical properties by using circular dichroism (right). A composite film dried without any magnetic field used as the control sample (reprint from Ref. [20]). (**d**) Schematic and circular dichroism of CNCs assembled into chiral nematic structure with rotation angle of 30° (left and right top, respectively). Schematic and circular dichroism of CNCs assembled into chiral nematic structure with rotation angle of 10° (left and right bottom, respectively) (reprint from Ref. [21]). (**e**) Top: Temporary vanishing of the Eu pattern on the CNC/polyacrylic acid composite upon acetone application, followed by reappearance of the pattern after rapid evaporation of the acetone. Bottom: chiral photoluminescence spectra of CNC–polyacrylic acid–Eu films at various wavelengths, recorded a) before and b) after water spraying (λₑₓ = 254 nm) (reprint from Ref. [22]). (**f**) Top: Photograph of the CNC/spiropyran composite film in its untreated state (left), after water application (middle), and following citric acid treatment (right), all under 365 nm UV light exposure. Bottom: the luminescence spectra of the composite film after application of water (left) and citric acid (right), respectively (reprint from Ref. [29]).

At fixed pitch distance, the CNC film selectively reflects the specific wavelength of the light, giving rise to the transmission of lights excepting the reflection wavelength. Also, fascinatingly, CNC film can selectively reflect the left-handed circular polarized lights. These features have opened its application to advanced electronics. For instance, motivated by the architecture of artificial intelligence systems that excel in efficient information integration and computational performance, a photonically active dielectric layer based on chiral nematic cellulose nanocrystals is integrated with printed p-type and n-type organic semiconductors to construct a multifunctional logic element (Figure 3a) [18]. These adaptive logic architectures are capable of producing discrete, tailored electrical output responses when exposed to light stimuli of varying photon energies, aligned with the photonic bandgaps of the active dielectric layer. The bifunctional configuration supports complex memory effects, enabled by reversible modulation of the photonic bandgap—through pitch variation in the chiral nematic structure—and differential light absorption across complementary organic semiconductor layers. This dynamic interplay results in a reconfigurable ternary logic behavior. As a proof of concept, this bio-derived, multivalued logic system represents a significant step toward low-power optical computing platforms, particularly for applications in integrated human–machine interface technology.

Additionally, Han and co-workers developed an optoelectronic synaptic device capable of modulating excitatory and inhibitory responses through light-mediated processes, achieved by employing humidity-responsive chiral nematic phases derived from well-characterized polysaccharide cellulose nanocrystals (Figure 3b) [19]. Environmentally driven modulation of the helical pitch in the chiral structure alters the polarization state of the helicoidal assembly, resulting in distinct hysteresis characteristics that govern both excitatory and inhibitory nonvolatile responses in bio-electrolyte-gated transistors. When voltage pulses are synergistically applied with chiral light stimulation, the artificial optoelectronic synapse is capable of modulating not only synaptic plasticity but also learning pathways and chromatic perception. These multifunctional, bio-derived synaptic field-effect transistors offer promising capabilities for advanced parallel neuromorphic computing and bioinspired robotic vision systems.

#### 3.1.2. Control of Reflected Lights

As an interesting example of research belonging to second category, Kim and co-workers reported weak and structured magnetic field gradients can produce left- and right-handed chiral and achiral regions due to the varying spatial arrangement of needle-shaped cellulose nanocrystals coated with magnetic materials (Figure 3c) [20]. The development of optically patterned thin films containing left- and right-handed chiral, as well as achiral regions, is attributed to vortices formed by local magnetic field gradients during the flow of liquid crystal suspensions. These patterns reflect the localized movement of magnetically coated nanocrystals during evaporation-driven assembly, illustrating how the competition between evaporation and magnetic field-induced flow shapes the twisted structural arrangement within the vortices. Simulations reveal that the inversion of twist is caused by the interaction between the direction and magnitude of depth-dependent magnetic gradients and the retreating evaporation front passing through magnetic gaps at the edges.

Additionally, Tsukruk and co-workers elegantly demonstrated the method for tuning the handedness of self-assembled CNCs through sequential printing of CNCs with twisted manner (Figure 3d) [21]. In this study, the sequential printing approach allows precise control over the chiroptical properties of twisted films by adjusting deposition parameters such as rotation angle, rotation direction, and film thickness. The resulting films, which exhibit distinct helical structures, display a range of unique optical activities with tunable circular dichroism (CD) peak position, sign, and intensity-enabled by the interplay of circular and linear birefringence. Additionally, the printing method is suitable for large-scale fabrication under ambient conditions, offering both accessibility and scalability. Furthermore, twisted printed films exhibit distinctive chiroptical properties. Unlike conventional thicker iridescent films formed by self-assembled CNCs, these films remain transparent due to having only 2 to 3 helical pitches. This exceptional transparency enables strong optical activity, including circular polarization with a remarkably high asymmetry factor for both transmitted and emitted light, while maintaining minimal light reflection. The notably high and tunable circularly polarized luminescence (CPL) asymmetry factors, along with their overall unique optical behavior, stem from the pre-designed helicity and handedness.

#### 3.1.3. Control of Emitted Lights

Meanwhile circular polarized luminescence can be generated if the emissive materials are incorporated into self-assembled CNCs. For instance, Kim and co-workers show the bio-synthetic light-emitting adhesive materials are derived from chiral nematic cellulose nanocrystal-polyelectrolyte-rare earth metal complexes and exhibit strong, universal adhesion to both hydrophilic and hydrophobic surfaces (Figure 3e) [22]. In this study, intense and dynamic photoluminescence with highly asymmetric and tunable right-handed circular polarization is achieved through minimal doping with europium, a rare earth metal, without affecting the adhesive strength or the original iridescent characteristics. The dissymmetry factor (g_lum_) quantifies the degree of circular polarization in the luminescence/emission of a chiral material. The highest |g_lum_| value of the sample was recorded as 0.46, which is meddle-high value among other CNC-based materials [23,24,25,26,27,28,29]. Interestingly, the photoluminescence can be temporarily suppressed by exposure to highly volatile acetone liquid, and it rapidly recovers upon dying, fully restoring its original emission intensity.

Kang and co-workers present durable and flexible light-emitting bio-photonic materials that show dynamic chiroptical activity (Figure 3f) [29]. The bio-based optically active materials, composed of CNCs arranged in a chiral nematic structure and doped with spiropyran, produce strong circularly polarized photoluminescence with a high |g_lum_| value of 0.61 [29]. Incorporating photochromic molecules into the chiral framework enables reversible phase shifts, allowing for switchable circularly polarized light emission with tunable handedness. This dynamic switching of polarization direction can be controlled in real time through either light exposure or changes in acidity.

### 3.2. Optical Sensors

**Figure 4:**
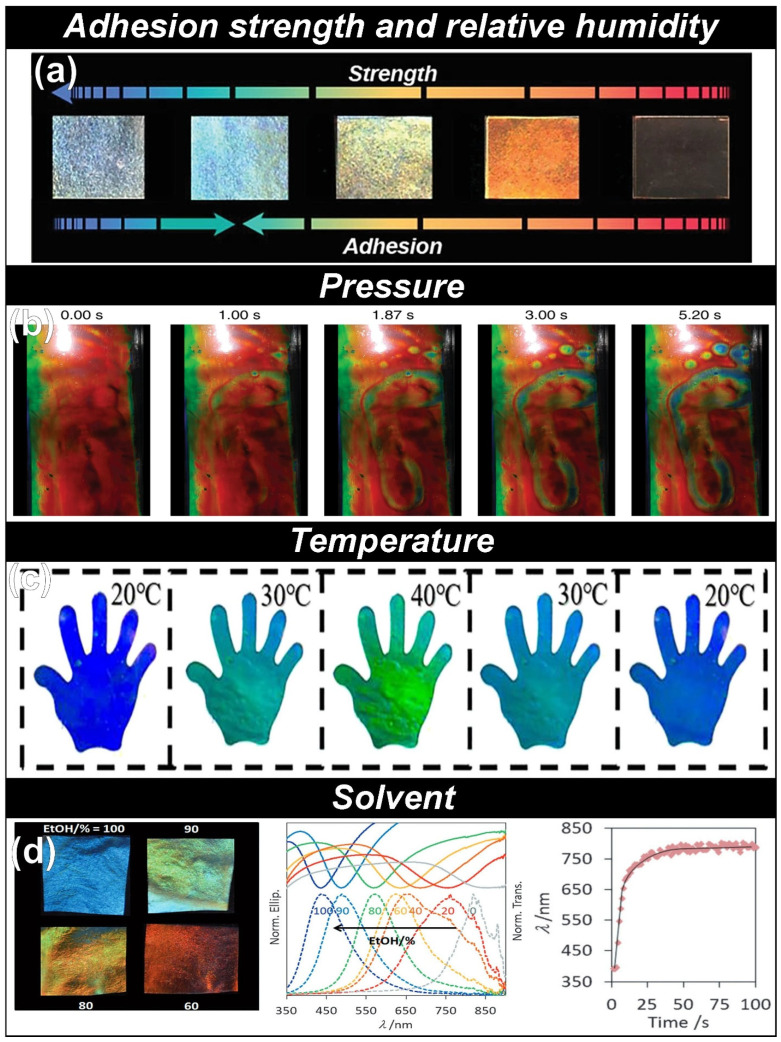
**(a**) Photograph of CNC/polyacrylamide/D-glucose photonic film that simultaneously changes its structural color and adhesion strength depending on the relative humidity. (reprint from Ref. [30]). (**b**) Photograph of participant’s footprint on laminated red hydroxypropyl cellulose film containing water as a function of standing time (reprint from Ref. [31]). (**c**) Optical visualization of thermally induced color transitions in palm-shaped hydroxypropyl cellulose/poly (acrylamide-co-acrylic acid)/carbon nanotube composite film (reprint from Ref. [32]). (**d**) (left) Photo and (middle) UV-Vis (solid line) and circular dichroism spectra (dashed line) of mesoporous photonic cellulose film swollen by ethanol (EtOH)/H_2_O mixtures with different ratios. The shift in the reflection peak of the mesoporous photonic cellulose film depending on the immersion time of the film in water (right) (reprint from Ref. [33]).

Sensing targets through visible color changes offers great benefits over other sensing methods such as electronic or electrochemical signal changes: (1) cost-effectiveness by sensing the target without measuring equipment and (2) quick and direct recognition of targets. Among diverse materials for colorimetric sensors, the choice of CNC films is of great option for realizing versatile as well as sustainable colorimetric sensors. Specifically, incorporating various functional material with CNCs has given rise to detection of myriads of targets. For instance, Kim and co-workers combined CNCs, D-glucose, and polyacrylamide (Figure 4a) [30]. In this study, CNCs functioned as a photonic component that can change colors depending on pitch distance. Next, D-glucose played a role as an adhesive and hygroscopic component that can absorb different amounts of water depending on the relative humidity changes. Lastly, polyacrylamide acted as a physical crosslinker, which can significantly improve the mechanical strength. The adaptive photonic systems, constructed from hydrogen-bonded chiral nematic architectures of CNCs, D-glucose, and polyacryamid, exhibit tunable adhesion properties governed by transitions between cohesive and interfacial failure modes under humid conditions. The dynamic optical behavior-manifested as color shifts spanning blue, red, and transparent states-is attributed to humidity-induced modulation of the helical pitch within the cholesteric structure. This pitch variation concurrently influences both mechanical robustness and interfacial adhesion. The resulting reversible tri-coupling of optical appearance, mechanical strength, and adhesion is harnessed to engineer advanced switchable bio-adhesives capable of rapid, remotely controlled transitions between adhesive and non-adhesive states. As potential applications, the authors utilized developed photonic adhesives as a visually trackable wound dressing for real-time healing assessment and colorimetric sensors integrated into respiratory masks for contamination detection.

In addition to sensing adhesion strength as well as relative humidity, CNC composites also can visibly sense pressure changes. For instance, Vignolini and co-workers reported the roll-to-roll fabrication of meter-scale hydroxypropyl cellulose (HPC) laminates achieved through a continuous coating and encapsulation process (Figure 4b) [31]. The pressure sensitivity of the water-encapsulated hydroxypropyl cellulose (HPC) laminates is quantitatively evaluated through optical analysis of pressure-induced hue shifts, detectable both by the human eye and digital imaging systems. Furthermore, the system demonstrates the capability to spatially and temporally resolve real-time pressure distributions, exemplified by capturing the dynamic evolution of a human footprint on the HPC surface. This represents the first large-area, cost-efficient fabrication of HPC-based stimuli-responsive photonic films capable of generating pressure maps readable by standard optical imaging devices. With incorporation of thermos-responsive polymer, poly (acrylamide-co-acrylic acid) (PACA), temperature change can be visualized (Figure 4c) [32]. Specifically, the PACA hydrogel is synthesized through extensive cross-linking of acrylamide and acrylic-based monomers. Upon heating, the intramolecular hydrogen bonds between acrylamide and acrylic acid moieties dissociate, allowing the polymer chains to establish hydrogen bonding interactions with surrounding water molecules. This results in water uptake and volumetric expansion of hydrogel. Conversely, as the temperature decreases, the hydrogen bonds between the polymer and water molecules are disrupted, facilitating the reformation of intramolecular hydrogen bonds within the polymer network. Consequently, hydrogel expels water and undergoes volumetric contraction. In this manner, with rising ambient temperature, the hydrogel scaffold undergoes thermal expansion, leading to an increase in the helical pitch of HPC. This structural change results in a red shift of the reflected wavelength, producing a visible color transition toward the red spectrum and vice versa.

Very fascinatingly, without incorporation of functional materials, self-assembled CNCs itself can visibly detect the various solvents. MacLachlan and co-workers reported a mesoporous cellulose-based material through a supramolecular co-templating approach (Figure 4d) [33]. Specifically, a composite material comprising cellulose nanocrystals and urea–formaldehyde resin self-assembles into a chiral nematic structure, which, upon alkaline treatment, gives rise to a chiral nematic mesoporous network of desulfated cellulose nanocrystals. These mesoporous photonic cellulose films exhibit rapid and reversible color changes in response to swelling, enabling their application in pressure sensing. Owing to their dynamic optical properties and structural versatility, these active mesoporous cellulose materials hold significant promises for applications in biosensing, photonics, functional membranes, chiral separation, and tissue engineering.

## 4. Concluding Remarks and Perspective

As illustrated above, CNCs have advanced optics and electronics thanks to their unique and fascinating properties: the occurrence of structural color, a chiral nematic structure, and high mechanical strength. However, there are remaining challenges to be solved for further advancing optics and electronics. For instance, CNC films often exhibit a highly polydisperse color profile and limited circularly polarized light reflection due to irregular arrangements and the formation of microscale aggregates with significant variation in pitch length and domain orientation [34,35]. To enhance structural uniformity and optical performance, several strategies have been explored, including the use of confined geometries, the regulation of humidity and temperature, the incorporation of external polymers, [36,37,38] and the application of magnetic or electric fields [39,40,41,42]. Increasing the length of the nanocrystals can expand the pitch of the chiral phase in dispersions and lower the critical concentration needed for chiral nematic ordering. Overall, the aspect ratio and surface functionalities of CNCs are critical factors in producing solid CNC films with strong optical anisotropy, distinct chirality, and tunable photonic bandgaps [43,44]. However, achieving precise control over pitch and structural uniformity across multiple length scales—essential for broadband reflection from UV to IR—remains a significant challenge 3]. Besides challenges such as the highly polydisperse color profile and limited circularly polarized light reflection, other bottlenecks still exist for CNCs in the field of optoelectronics. For instance, research utilizing CNCs as chiral optical components has primarily been conducted using wood pulp as the source of CNCs. Consequently, there is a significant lack of fundamental knowledge regarding the chiroptical properties of CNCs derived from sources other than wood pulp (e.g., the chiroptical properties of CNCs produced from rice husks remain unexplored to the best of our knowledge). Uncovering the chiral optical properties of CNCs from other sources than wood pulp could enable further advancements in optoelectronics. To achieve this goal, multidisciplinary research is essential, particularly collaborative efforts between materials science/chemistry and biomass process engineering. Dynamic phase changes like transitions between different ordered states or between ordered and disordered phases are key for designing soft nanomaterials that can undergo dramatic yet predictable changes in function. However, only a limited number of examples have shown how to build stable, large-scale chiral bio-photonic materials with controllable left- or right-handedness, guided by a testable physical model and a comprehensive understanding of their intricate assembly processes. The dynamics and reversible handedness of circular polarized light generated from CNCs still have not been reported yet to the best of our knowledge. To realize this goal, the combination of CNC with other chiral materials and/or elastomeric materials will be needed, possibly with engineering techniques such as lithography, printing, and/or thin-film deposition.

We expect the wearable sensors can be further advanced by employing CNCs. Wearable sensors represent a transformative approach for the real-time monitoring and transmission of personal health data [45]. However, the achievement of high-performance wearable sensors still faces major challenges, including highly selective sensing for circular polarized lights, selective interaction with chiral molecules, and maintaining mechanical durability during repeated use. To address these hurdles, the incorporation of chiral materials has emerged as a promising and innovative strategy, offering both practical and efficient solutions [19,46,47,48,49,50,51,52,53]. Despite this, research on wearable sensors utilizing the chiral properties of CNC has rarely been reported. Thus, by achieving high chiroptical properties such as a narrow photonic band gap and high anisotropic factors, CNC could play as a key role for the development of advanced high-performance wearable sensors.

## References

[1] Nepal D., Kang S., Adstedt K.M., Kanhaiya K., Bockstaller M.R., Brinson L.C., Buehler M.J., Coveney P.V., Dayal K., El-Awady J.A. (2023). Hierarchically structured bioinspired nanocomposites. Nat. Mater..

[2] Kim M., Chung H. (2017). Photo-responsive bio-inspired adhesives: Facile control of adhesion strength via a photocleavable crosslinker. Polym. Chem..

[3] Xiong R., Grant A.M., Ma R., Zhang S., Tsukruk V.V. (2018). Naturally-derived biopolymer nanocomposites: Interfacial design, properties and emerging applications. Mater. Sci. Eng..

[4] Wu X., Shi Z., Fu S., Chen J., Berry R.M., Tam K.C. (2016). Strategy for synthesizing porous cellulose nanocrystal supported metal nanocatalysts. ACS Sustain. Chem. Eng..

[5] Walters C.M., Matharu G.K., Hamad W.Y., Lizundia E., MacLachlan M.J. (2021). Chiral nematic cellulose nanocrystal/germania and carbon/germania composite aerogels as supercapacitor materials. Chem. Mater..

[6] Walters C.M., Adair K.R., Hamad W.Y., MacLachlan M.J. (2020). Synthesis of chiral nematic mesoporous metal and metal oxide nanocomposites and their use as heterogeneous catalysts. Eur. J. Inorg. Chem..

[7] Li T., Chen C., Brozena A.H., Zhu J.Y., Xu L., Driemeier C., Dai J., Rojas O.J., Isogai A., Wagberg L. (2021). Developing fibrillated cellulose as a sustainable technological material. Nature.

[8] Kalashnikova O., Pankova E., Sukhikh S., Babich O., Samusev I., Tcibulnikova A., Ivanova S., Kriger O. (2024). Production of bacterial cellulose using a symbiotic consortium of bacteria and yeast on soybean molasses medium. LWT-Food Sci. Technol..

[9] Wang Y., Liu H., Wang Q., An X., Ji X., Tian Z., Liu S., Yang G. (2023). Recent advances in sustainable preparation of cellulose nanocrystals via solid acid hydrolysis: A mini-review. Int. J. Biol. Macromol..

[10] Moon R.J., Martini A., Nairn J., Simonsen J., Youngblood J. (2011). Cellulose nanomaterials review: Structure, properties and nanocomposites. Chem. Soc. Rev..

[11] Riccardi C.M., Kasi R.M., Kumar C.V., Challa V.K. (2017). Chapter Nineteen—Nanoarmoring of Enzymes by Interlocking in Cellulose Fibers with Poly(Acrylic Acid). Methods in Enzymology.

[12] Adstedt K., Popenov E.A., Pierce K.J., Xiong R., Geryak R., Cherpak V., Nepal D., Bunning T.J., Tsukruk V.V. (2020). Chiral cellulose nanocrystals with intercalated amorphous polysaccharides for controlled iridescence and enhanced mechanics. Adv. Funct. Mater..

[13] Korolovych V.F., Cherpak V., Nepal D., Ng A., Shaikh N.R., Grant A., Xiong R., Bunning T.J., Tsukruk V.V. (2018). Cellulose nanocrystals with different morphologies and chiral properties. Polymer.

[14] Tran A., Boott C.E., MacLachlan M.J. (2020). Understanding the self-assembly of cellulose nanocrystals—Toward Chiral photonic materials. Adv. Mater..

[15] Rusli R., Eichhorn S.J. (2008). Determination of the stiffness of cellulose nanowhiskers and the fiber-matrix interface in a nanocomposite using Raman spectroscopy. Appl. Phys. Lett..

[16] Zhang X., Xiong R., Kang S., Yang Y., Tsukruk V.V. (2020). Alternating stacking of nanocrystals and nanofibers into ultrastrong chiral biocomposite laminates. ACS Nano.

[17] Zhang X., Liu Y., Han J., Kivshar Y., Song Q. (2022). Chiral emission from resonant metasurfaces. Science.

[18] Han M.J., Kim M., Tsukruk V.V. (2022). Multivalued logic for optical computing with photonically enabled chiral bio-organic structures. ACS Nano.

[19] Han M.J., Tsukruk V.V. (2023). Trainable bilingual synaptic functions in bio-enabled synaptic transistors. ACS Nano.

[20] Kim M., Jeon J., Pierce K., Bukharina D., Choi W., Choi J., Nepal D., McConney M.E., Bunning T.J., Tsukruk V.V. (2024). Magneto-Responsive Chiral Optical Materials: Flow-Induced Twisting of Cellulose Nanocrystals in Patterned Magnetic Fields. ACS Nano.

[21] Bukharina D., Southard L., Dimitrov B., Brackenridge J.A., Kang S., Min P., Wang Y., Nepal D., McConney M.E., Bunning T.J. (2024). Left and Right—Handed Light Reflection and Emission in Ultrathin Cellulose Nanocrystals Films with Printed Helicity. Adv. Funct. Mater..

[22] Kim M., Lee H., Snipes R.T., Han M.J., Tsukruk V.V. (2022). Co-Assembly of Biosynthetic Chiral Nematic Adhesive Materials with Dynamic Polarized Luminescence. Small.

[23] Li W., Xu M., Ma C., Liu Y., Zhou J., Chen Z., Wang Y., Yu H., Li J., Liu S. (2019). Tunable Upconverted Circularly Polarized Luminescence in Cellulose Nanocrystal Based Chiral Photonic Films. ACS Appl. Mater. Interfaces.

[24] Xiong R., Yu S., Smith M.J., Zhou J., Krecker M., Zhang L., Nepal D., Bunning T.J., Tsukruk V.V. (2019). Self-Assembly of Emissive Nanocellulose/Quantum Dot Nanostructures for Chiral Fluorescent Materials. ACS Nano.

[25] Chekini M., Prince E., Zhao L., Mundoor H., Smalyukh I.I., Kumacheva E. (2020). Chiral Carbon Dots Synthesized on Cellulose Nanocrystals. Adv. Opt. Mater..

[26] He J., Bian K., Li N., Piao G. (2019). Generation of full-color and switchable circularly polarized luminescence from nonchiral dyes assembled in cholesteric cellulose films. J. Mater. Chem. C.

[27] Zheng H., Li W., Li W., Wang X., Tang Z., Zhang S.X.A., Xu Y. (2018). Uncovering the Circular Polarization Potential of Chiral Photonic Cellulose Films for Photonic Applications. Adv. Mater..

[28] Xu M., Wu X., Yang Y., Ma C., Li W., Yu H., Chen Z., Li J., Zhang K., Liu S. (2020). Designing Hybrid Chiral Photonic Films with Circularly Polarized Room-Temperature Phosphorescence. ACS Nano.

[29] Kang S., Li Y., Bukharina D., Kim M., Lee H., Buxton M.L., Han M.J., Nepal D., Bunning T.J., Tsukruk V.V. (2021). Bio-Organic Chiral Nematic Materials with Adaptive Light Emission and On-Demand Handedness. Adv. Mater..

[30] Kim M., Lee H., Krecker M.C., Bukharina D., Nepal D., Bunning T.J., Tsukruk V.V. (2021). Switchable photonic bio-adhesive materials. Adv. Mater..

[31] Liang H.L., Bay M.M., Vadrucci R., Barty-King C.H., Peng J., Baumberg J.J., De Volder M.F.L., Vignolini S. (2018). Roll-to-roll fabrication of touch-responsive cellulose photonic laminates. Nat. Commun..

[32] Zhang Z., Chen Z., Wang Y., Zhao Y. (2020). Bioinspired conductive cellulose liquid-crystal hydrogels as multifunctional electrical skins. Proc. Natl. Acad. Sci. USA.

[33] Giese M., Blusch L.K., Khan M.K., Hamad W.Y., MacLachlan M.J. (2014). Responsive mesoporous photonic cellulose films by supramolecular cotemplating. Angew. Chem. Int. Ed..

[34] O’Keeffe O., Wang P.X., Hamad W.Y., MacLachlan M.J. (2019). Boundary Geometry Effects on the Coalescence of Liquid Crystalline Tactoids and Formation of Topological Defects. J. Phys. Chem. Lett..

[35] Dumanli A.G., van der Kooij H.M., Kamita G., Reisner E., Baumberg J.J., Steiner U., Vignolini S. (2014). Digital color in cellulose nanocrystal films. ACS Appl. Mater. Interfaces.

[36] Parker R.M., Frka-Petesic B., Guidetti G., Kamita G., Consani G., Abell C., Vignolini S. (2016). Hierarchical self-assembly of cellulose nanocrystals in a confined geometry. ACS Nano.

[37] Yao K., Meng Q., Bulone V., Zhou Q. (2017). Flexible and responsive chiral nematic cellulose nanocrystal/poly (ethylene glycol) composite films with uniform and tunable structural color. Adv. Mater..

[38] Lee C.C., Grenier C., Meijer E., Schenning A.P. (2009). Preparation and characterization of helical self-assembled nanofibers. Chem. Soc. Rev..

[39] Zhao T.H., Parker R.M., Williams C.A., Lim K.T.P., Frka-Petesic B., Vignolini S. (2018). Printing of Responsive Photonic Cellulose Nanocrystal Microfilm Arrays. Adv. Funct. Mater..

[40] Natarajan B., Emiroglu C., Obrzut J., Fox D.M., Pazmino B., Douglas J.F., Gilman J.W. (2017). Dielectric characterization of confined water in chiral cellulose nanocrystal films. ACS Appl. Mater. Interfaces.

[41] Frka-Petesic B., Guidetti G., Kamita G., Vignolini S. (2017). Controlling the Photonic Properties of Cholesteric Cellulose Nanocrystal Films with Magnets. Adv. Mater..

[42] Paajanen A., Ceccherini S., Maloney T., Ketoja J.A. (2019). Chirality and Bound Water in the Hierarchical Cellulose Structure. Cellulose.

[43] Liu D., Wang S., Ma Z., Tian D., Gu M., Lin F. (2014). Structure–color mechanism of iridescent cellulose nanocrystal films. RSC Adv..

[44] Bardet R., Belgacem N., Bras J. (2015). Flexibility and color monitoring of cellulose nanocrystal iridescent solid films using anionic or neutral polymers. ACS Appl. Mater. Interfaces.

[45] Bandodkar A.J., Wang J. (2014). Non-invasive wearable electrochemical sensors: A review. Trends Biotechnol..

[46] Zhong B., Qin X., Xu H., Liu L., Li L., Li Z., Cao L., Lou Z., Jackman J.A., Cho N.-J. (2024). Interindividual-and blood-correlated sweat phenylalanine multimodal analytical biochips for tracking exercise metabolism. Nat. Commun..

[47] Yang X.L., Yang Z.Y., Shao R., Guan R.F., Dong S.L., Xie M.H. (2023). Chiral MOF derived wearable logic sensor for intuitive discrimination of physiologically active enantiomer. Adv. Mater..

[48] Wu M., Wang Y., Gao S., Wang R., Ma C., Tang Z., Bao N., Wu W., Fan F., Wu W. (2019). Solution-synthesized chiral piezoelectric selenium nanowires for wearable self-powered human-integrated monitoring. Nano Energy.

[49] Hu T., Pan T., Guo D., Xiao Y., Li F., Gao M., Huang Z., Zhu J., Cheng T., Lin Y. (2023). Omnidirectional configuration of stretchable strain sensor enabled by the strain engineering with chiral auxetic metamaterial. ACS Nano.

[50] Paul S., Barman S., Pal A., Mukherjee A., Ghosh S., Datta A. (2023). Piezoelectric Micropower Harvester from Supramolecular Assembly of Chiral Ambipolar Chromophores. Chem. Mater..

[51] An L.-C., Zhao C., Zhao Y., Zhang Y., Li K., Stroppa A., Li W., Bu X.-H. (2023). Chiral 1D hybrid metal halides with piezoelectric energy harvesting and sensing properties. Small Struct..

[52] Dai H., Hong R., Ma Y., Cheng X., Zhang W. (2023). A Subtle Change in the Flexible Achiral Spacer Does Matter in Supramolecular Chirality: Two-Fold Odd-Even Effect in Polymer Assemblies. Angew. Chem. Int. Ed..

[53] Wang L., Xue Y., Cui M., Huang Y., Xu H., Qin C., Yang J., Dai H., Yuan M. (2020). A chiral reduced-dimension perovskite for an efficient flexible circularly polarized light photodetector. Angew. Chem..

